# Survey of Grain Boundary Energies in Tungsten and Beta-Titanium at High Temperature

**DOI:** 10.3390/ma15010156

**Published:** 2021-12-26

**Authors:** Hong He, Shangyi Ma, Shaoqing Wang

**Affiliations:** 1 Shenyang National Laboratory for Materials Science, Institute of Metal Research, Chinese Academy of Sciences, Shenyang 110016, China; hhe16s@imr.ac.cn (H.H.); shyma@imr.ac.cn (S.M.); 2 School of Materials Science and Engineering, University of Science and Technology of China, Shenyang 110016, China

**Keywords:** grain boundary energy, molecular dynamics, body-centered cubic metals, high temperature

## Abstract

Heat treatment is a necessary means to obtain desired properties for most of the materials. Thus, the grain boundary (GB) phenomena observed in experiments actually reflect the GB behaviors at relatively high temperature to some extent. In this work, 405 different GBs were systematically constructed for body-centered cubic (BCC) metals and the grain boundary energies (GBEs) of these GBs were calculated with molecular dynamics for W at 2400 K and *β*-Ti at 1300 K and by means of molecular statics for Mo and W at 0 K. It was found that high temperature may result in the GB complexion transitions for some GBs, such as the Σ11{332}{332} of W. Moreover, the relationships between GBEs and sin(*θ*) can be described by the functions of the same type for different GB sets having the same misorientation axis, where *θ* is the angle between the misorientation axis and the GB plane. Generally, the GBs tend to have lower GBE when sin(*θ*) is equal to 0. However, the GB sets with the <110> misorientation axis have the lowest GBE when sin(*θ*) is close to 1. Another discovery is that the local hexagonal-close packed *α* phase is more likely to form at the GBs with the lattice misorientations of 38.9°/<110>, 50.5°/<110>, 59.0°/<110> and 60.0°/<111> for *β*-Ti at 1300 K.

## 1. Introduction

The structure and properties of grain boundaries (GBs) play a decisive role on the performances of polycrystal materials [1]. Owing to the significance of GBs in polycrystal materials, the GB engineering was proposed in 1980s [2]. The GB engineering is an approach to improve the properties of materials by controlling the grain boundary character distribution (GBCD), mainly by increasing the proportion of the so-called special GBs [2,3]. In recent decades, many works have been devoted to study the GBCD and microstructure evolution of polycrystal materials [3,4] as well as the grain boundary energy distribution (GBED) [5,6,7,8,9,10], and a consensus was reached that the GBCD and GBED are negatively correlated with each other on average. The grain boundary energy (GBE) is one of the fundamental structure-dependent properties of GBs and is not only related to GBCD, but also affect the behaviors of GBs [2]. The GB phenomena, such as corrosion and segregation, are more likely to take place at the GBs with higher GBE, while the GBs with lower GBE show a stronger resistance to the abovementioned GB phenomena [4]. Therefore, the better understanding of the microstructure and GBE of the GBs, the more beneficial it will be for the material design based on the GB engineering.

The tri-crystal and thermal groove methods are two main methods to measure the GBEs in experiments [4,11], but the high cost and low accuracy limit the wide application of the two methods. With the development of the computing methods and power, the atomic simulation has become an important method to evaluate the GBEs of various GBs, as well as exploring the explicit GB structures at atomic scale.

In recent three decades, many works have been done to calculate the GBEs and explore the relationships between GBEs and geometric factors [4,12,13,14,15,16,17,18,19,20,21,22,23,24,25], such as the GB plane and the ∑ value which is the reciprocal density of coincidence sites and is equal to the cell volume ratio of coincidence site lattice and crystal lattice [26]. Due to the complex nature of the GB structures defined by five parameters (three to describe the misorientation between two grains and two to define the GB plane orientation [1]), most of the previous researches focused on the special GBs, such as the symmetric tilt grain boundaries [12,13,14,27,28] (STGBs) and the twist grain boundaries [15,17] (TWGBs), which limits the complete description of the relationships between GBEs and GB microstructures. In the past few years, with the development of the method to construct GBs [18,19,21,25], the studies of GB properties have been extended to the large GB sets containing arbitrary GBs of some face-centered cubic (FCC) [6,21,22,23,24,29,30] and body-centered cubic (BCC) metals [20,25]. All these calculations indicate that there is a simple linear relationship of GBEs between different metals having the same crystal structure. Furthermore, the functions that can quantitatively describes the GBE variations in the 5-D space of the five parameters defining GB structures were proposed for some FCC [24,31] and BCC [32,33] metals, based on the large GB sets. However, any variable of the five parameters is difficult to extract from the fitted functions for arbitrary GBs. Thus, the relationships between GBEs and geometric factors, if they exist, were still elusive for FCC and BCC metals and it is still a challenge to describe such relationships by some simple functions.

To our knowledge, most of the theoretical studies of GB properties are based on molecular statics (MS) or density functional theory (DFT) method, by which the temperature effects are not considered. However, in most applications, the heat treatment is a necessary process to obtain the desired properties of metals and alloys. The GB microstructures observed in experiments and the corresponding GB properties actually reflect the temperature effects on GBs to some extent [3,34], because the change of temperature may lead to the transition of GB complexions, which is important to the properties of polycrystals [35,36,37]. The latest atomistic simulations reveal that there are several different metastable microstructures for the same GB and the further MD simulations showed that the stable structures at high temperature are related to these metastable structures for FCC [38,39] and BCC [40,41] metals. Recently, the coexistence of two different GB complexions in pure copper were observed experimentally [42], which gave the direct evidence for the transition of GB complexions in pure metals. Therefore, the systematic simulation of GB properties at high temperature is quite necessary to extend the knowledge of GBs and to bridge the relationship between simulations and experiments.

In this work, a GB set containing 405 different GBs for BCC metals of W, Mo, and *β*-Ti was built, and the GBEs of W and *β*-Ti at high temperature were calculated by molecular dynamics (MD) method. The selection of metal W was motivated by its stability with BCC structure until melting point and its application in nuclear fusion reactors at high temperature [43]. The metal *β*-Ti was chosen because the BCC *β*-Ti can only be stabilized between 1155 (allotropic transformation temperature, *T*_transf_) and 1943 K (melting temperature, *T*_melt_) [44]. Meanwhile, the GBEs of Mo and W at 0 K were also calculated for comparison. On the basis of the calculations, the effects of temperature on GBEs and the relationships between GBEs and geometric factors were explored. Besides, the geometric characteristics of the special GBs with lower GBE were also investigated in this work.

## 2. Computational Methods

### 2.1. Construction of GB Models

**Figure 1:**
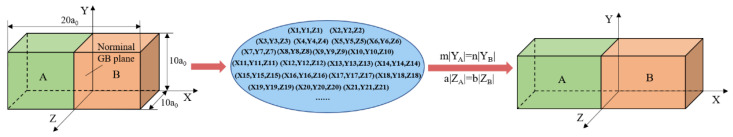
The grain boundary (GB) models generation process.

The idea proposed by Olmsted et al. [21] was adopted to construct a GB set containing arbitrary types of GBs by specifying the limitation size of the computational cell. Figure 1 schematically shows how to construct the GB models. Firstly, create an orthogonal box with the size of 20*a*_0_ (X) × 10*a*_0_ (Y) × 10*a*_0_ (Z), where *a*_0_ is the lattice constant, X is the axis perpendicular to the GB plane, and Y and Z are the axes parallel to the GB plane. The plane of *x* = 0 is taken as the nominal GB plane. Then, list all possible crystals that the periodic lengths along *X*, Y, and Z axes are no more than 10*a*_0_. Every possible crystal here is defined by the crystal orientations along X, Y, and Z axes. Thirdly, select two crystals from the above crystals and connect them to generate a GB. The two selected crystals were limited to those that the periodic lengths along Y and Z axes can be matched, namely *m*|*Y*_A_| = *n*|*Y*_B_| and *a*|*Z*_A_| = *b*|*Z*_B_|, where |*Y*_A_| and |*Z*_A_| are the periodic lengths along Y and Z axes of grain A, |*Y*_B_| and |*Z*_B_| are the periodic lengths along Y and Z axes of grain B, and *m*, *n*, *a*, and *b* are all integers. Then, repeat the same process to obtain all possible GBs. Next, delete all duplicate GBs according to the symmetry elements of BCC structures. Fifthly, characterize the GBs with the geometric factors, including the ∑ value, the misorientation axis and angle, the tilt axis and angle, and the twist axis and angle. Finally, a GB set containing 405 GBs was obtained, which consists of 83 STGBs, 294 asymmetric tilt grain boundaries (ATGBs), 172 TWGBs, and 21 mixed grain boundaries (MGBs). Note that 165 GBs are both twist and tilt GBs, depending on the choice of rotation axis. Besides, all GBs have 80 different misorientations and the ∑ value ranges from 3 to 387.

### 2.2. Computational Details

The LAMMPS code [45] was utilized to compute the GBEs of Mo and W at 0 K by MS simulations and the GBEs of W at 2400 K and *β*-Ti at 1300 K by MD simulations. 

In this work, the periodic boundary conditions (PBCs) along X, Y, and Z axes were adopted for all atomic simulations. Thus, the GBE is defined by the following expression:

(1) $$\gamma_{\mathrm{GB}}=\frac{E_{\mathrm{GB}}-NE_{\mathrm{bulk}}}{2A},$$

where *γ*_GB_ labels the GBE, *E*_GB_ is the total potential energy of the GB system, *E*_bulk_ is the potential energy of each atom in a bulk system at the temperature same as the GB system, *N* is the total number of the atoms in the GB system, and *2A* represents the total area of the two GB planes in the computational supercell.

#### 2.2.1. MS Simulations of GBs

The conjugate-gradient method implemented in the LAMMPS code [45] was used for the MS calculations of a serial of GB systems of Mo and W at 0 K as well as their bulk systems. The adopted potentials for Mo and W are the modified-embedded-atom method (MEAM) potential developed by Park [46] and the embedded-atom method (EAM) potential developed by Zhou [47], respectively. The accuracy of the EAM potential developed by Zhou to calculate the GBEs of W had been roughly verified by experiments that the calculated GBED are inversely correlated with the experimentally observed GBCD. The length of the computational cell along X axis, which is perpendicular to the GB plane, was limited to no less than 30*a*_0_ to avoid the interactions between two boundaries in the computational supercell. 

The *γ*-surface method [20,21,48,49,50], a commonly used method to predict the GB microstructures of the ground state, was adopted to calculate the GBEs of Mo and W by MS simulations. For every GB model constructed by connecting two perfect crystals, the crystal on one side of the GB plane was shifted relative to the other on the grid which is parallel to the GB plane and has the size of *a*_0_/9 × *a*_0_/9, and then the corresponding 81~29, 241 initial microscopic structures were generated and minimized. The microscopic structure with the minimum GBE was considered as the ground state of the GB and the corresponding GBE was regarded as the actual GBE of the GB.

#### 2.2.2. MD Simulations of GBs

The GBEs of W and *β*-Ti at high temperature were calculated by MD simulations with the EAM potential developed by Zhou [47] and the EAM/FS potential developed by Mendelev [51], respectively. Given that *β*-Ti is only stable between 1155 (*T*_transf_) and 1943 K (*T*_melt_) [44] and the ordered arrangement of atoms at GBs will be destroyed when the temperature is close to the melting point [52], the GBs of *β*-Ti were dynamically relaxed at 1300 K (0.67*T*_melt_). The choice of the simulation temperature is also related to the annealing temperature of GB engineering, which is generally 0.6~0.8 of the absolute melting temperature [34]. Referring to the simulation temperature of *β*-Ti, the dynamic relaxations of the GBs were performed at 2400 K (0.65*T*_melt_) for W. The length along X axis of the computational cell is greater than 64*a*_0_ to avoid GB interactions.

The dynamic relaxations of the GBs were performed in the NPT ensemble with the timestep of 2 *fs*. Every GB system was relaxed for 14 *ns* and the time average of the total potential energy between 12 and 14 *ns* was adopted to calculate the GBEs of the GBs at high temperature.

## 3. Results and Discussion

### 3.1. The Relationships between GBEs and Geometric Factors

**Figure 2:**
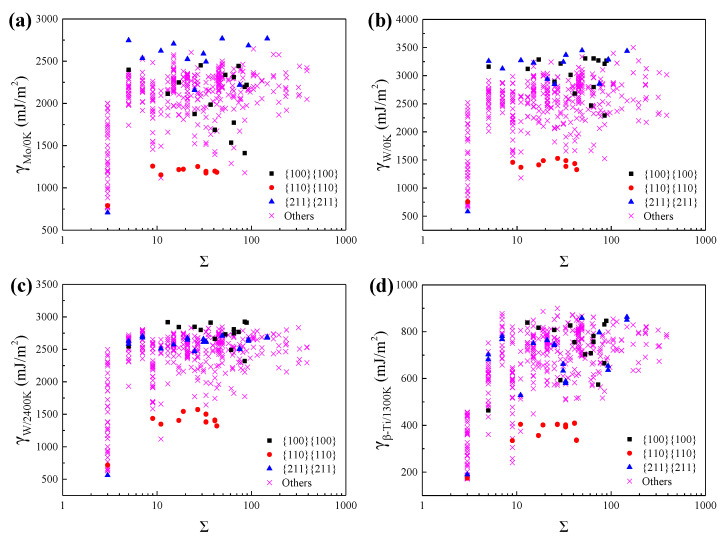
The calculated grain boundary energies (GBEs) of (**a**) Mo at 0 K, (**b**) W at 0 K, (**c**) W at 2400 K, and (**d**) *β*-Ti at 1300 K as a function of the ∑ value. The black squares, red dots, blue triangles, and purple crosses correspond to the GBs with the GB planes of {100}{100}, {110}{110}, {211}{211}, and others, respectively.

The coincidence site lattice (CSL) has been widely used to describe the lattice misorientation between two adjacent grains and the associated ∑ notation is the reciprocal density of CSL. Figure 2 shows the variation of GBE with ∑ value for Mo at 0 K, W at 0 K, W at 2400 K, and *β*-Ti at 1300 K. As shown in Figure 2, the ∑3{211}{211} and ∑9{110}{110} have almost the lowest GBE of the ∑3 and the ∑9 GBs, respectively. Besides, the GBs with the GB plane of {110}{110} also have lower GBE of all GBs. Thus, it was speculated that the populations of these special GBs are relatively high in the BCC metals. Actually, the simulated GBEDs of these special GBs in Mo, W, and *β*-Ti are similar to the previously simulated and measured GBEDs of these special GBs in Fe [7,53], Mo [20] and W [9,54]. Besides, it was observed experimentally that for GBs in Fe [7,53] and W [9,54], the ∑3{211}{211} and the ∑9{110}{110} have the highest population of the ∑3 GBs and the ∑9 GBs, respectively, and the GBs with the GB plane of {110}{110} also have relatively high populations. Accordingly, it was concluded that the calculated GBEDs of these special GBs for Mo, W, and *β*-Ti are inversely correlated with the experimentally observed GBCDs of these special GBs for Mo, W, and Fe. This verifies the accuracy of the methods used in this work to calculate the GBEs to some extent.

**Figure 3:**
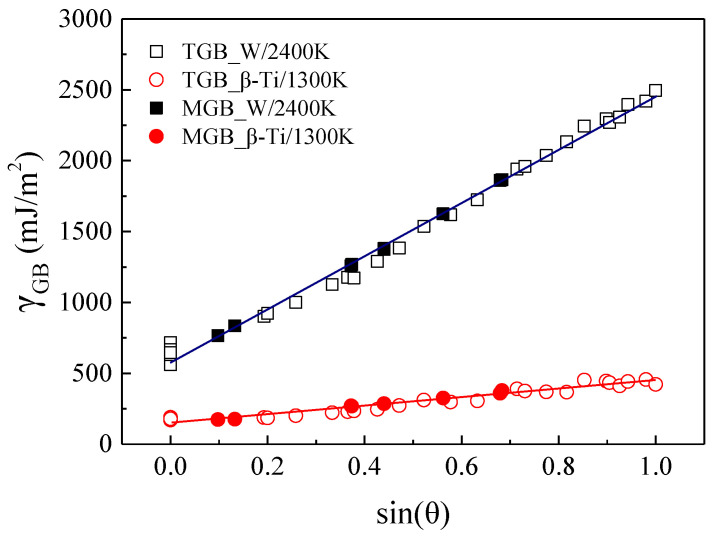
The GBEs of ∑3 GBs as a function of sin(*θ*). The *θ* is the angle between the misorientation axis and the GB plane.

As shown in Figure 2, there could be multiple different GBs and the corresponding GBEs may span a broad spectrum when the ∑ value is specified, such as the ∑3 GBs. Therefore, there is no direct relationship between GBEs and the ∑ values, which has been reported by Olmsted and Ratanaphan et al. [20,21]. The difference of the GB plane orientations is the main reason resulting in the existence of many different GBs when the ∑ value has been specified. Therefore, the GB plane orientations play a critical role in determining the GBEs of the GBs. The relationships between GBEs and the GB plane orientations have been investigated in our previous work [55] that the relationships between GBEs and sin(*θ*), where *θ* is the angle between the misorientation axis and the GB plane, can be described by some simple functions when the lattice misorientation is specified. However, the investigation focused only on the tilt grain boundaries (TGBs) of W and *β*-Ti. In order to extend such correlations to a more general applicability, the relationships between GBEs and sin(*θ*) for GBs having the same lattice misorientation and including all types of GBs were explored. It was found that the relationships between GBEs and sin(*θ*) can still be described by some simple functions, such as the ∑3 GBs including 20 TGBs and 8 MGBs, as shown in Figure 3. The relationships between GBEs of these ∑3 GBs and sin(*θ*) can be fitted by the following linear expressions:

(2) $$\gamma_{\mathrm{WHT}}=579+1868\sin\left(\theta \right),$$

(3) $$\gamma_{\beta \_\mathrm{Ti}}=153+293\sin(\theta ),$$

where *γ*_WHT_ and *γ_β_*_-Ti_ represent the GBEs of W at 2400 K and *β*-Ti at 1300 K, respectively. The slopes of *γ*_WHT_ and *γ_β_*_-Ti_, 1868 and 293, for ∑3 GBs including TGBs and MGBs are close to the slopes of *γ*_WHT_ and *γ_β_*_-Ti_, 1900 and 298, for ∑3 TGBs, respectively [55]. Besides, it is obviously seen from Figure 3 that the distribution of the solid points regarding to MGBs of W at 2400 K and *β*-Ti at 1300 K generally follow the correspondingly fitted lines. Therefore, it was concluded that the relationship between GBEs and sin(*θ*) can be described by a simple function for GBs having the same lattice misorientation, which is irrespective of the type of GBs. This makes it possible to predict or explain the preferred GB plane orientation for GBs with the same lattice misorientation.

There is also a special set of GBs with {110}{110} GB planes, the GBEs of which are lower and independent of misorientation lattice, as shown in Figure 2. This can be attributed to the special characteristics of the {110} planes which have the highest atomic density per unit area and the lowest surface energy in BCC metals [56].

### 3.2. The High Temperature Effects on GBs

**Figure 4:**
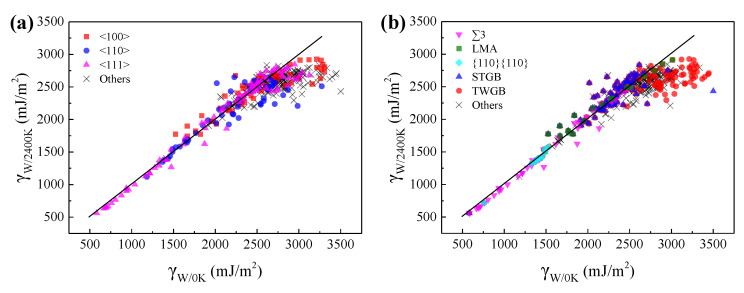
The comparison of GBEs of W at 0 and 2400 K. Every point in (**a**,**b**) corresponds a GB and the horizonal and vertical coordinates of every point correspond to the GBEs of the GB in W at 0 and 2400 K, respectively. The points in (**a**) are classified according to the misorientation axis of GBs, while the points in (**b**) are classified into ∑3 GBs, low misorientation angle (LMA) GBs, {110}{110} GBs, symmetric tilt grain boundaries (STGBs), twist grain boundaries (TWGBs), and other GBs. The black lines with slope of 1 were drawn for comparison.

To explore the high temperature effects on the GBEs of GBs, the GBEs of W at 2400 K were calculated and compared with the GBEs of W at 0 K, as shown in Figure 4.

The GBEs fluctuate within a very small range as the temperature increase from 0 to 2400 K for most of the GBs, except for the special ones with high calculated GBE at 0 K. Figure 4 shows that these special GBs tend to have lower GBE at 2400 K. For example, the GBEs of ∑33{211}{211} in W at 0 and 2400 K are 3369 and 2620 mJ·m^−2^, respectively. Our further calculations show that the GBE gap of ~749 mJ·m^−2^ results from their different GB microstructures. Due to the limitation of the *γ*-surface method, it is not guaranteed that every obtained GB microstructure of W at 0 K is in a stable state with the lowest GBE. Actually, some obtained GBs are in a metastable state with high GBE. The dynamic relaxation of these metastable GBs at high temperature can help us to obtain the more stable states of lower GBEs at 0 K for these special GBs. Compared with the GB microstructures and energies obtained by the *γ*-surface method, when the stable GB microstructures at high temperature were adopted as the original GB structures to relax at 0 K, the more stable states with lower GBEs will be obtained for these metastable GBs. For example, the calculated GBE of the ∑33{211}{211} at 0 K is 2843 mJ·m^−2^ by adopting the stable GB microstructure at 2400 K as the original structure. As shown in Figure 4, these special GBs are mostly the TWGBs having high calculated GBE at 0 K and the GBs with high crystal orientation index of the misorientation axis, which are more complexed in GB structure. Therefore, it was concluded that the *γ*-surface method is not always appropriate for predicting the GBEs and microstructures of GBs, especially for those having complexed GB structures.

**Figure 5:**
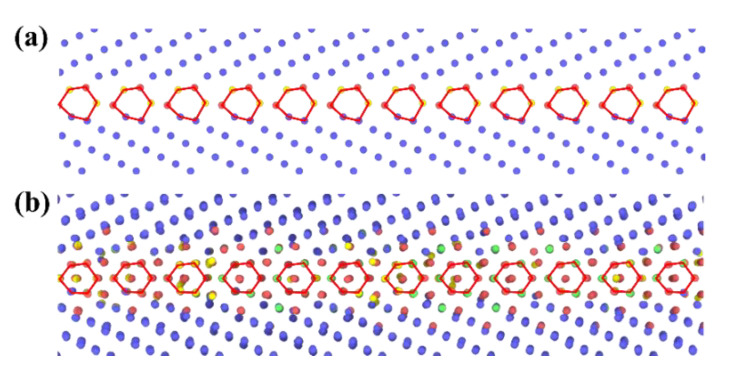
The GB microstructures of Σ11{332}{332} for (**a**) W at 0 and (**b**) W at 2400 K. The structure units of the GBs are highlighted by red polygons. The atoms arranged in body-centered cubic (BCC), face-centered cubic (FCC), hexagonal close-packed (HCP), and other patterns are colored by bule, green, red, and yellow, respectively.

In addition, high temperature may result in the transition of GB complexions [36,39,40,42,57,58,59,60]. The term “GB complexion” refers to the thermodynamically stable state of a GB [36]. The GB complexion transitions have also been found for some GBs of W in this work, accompanied with the change of GBE. Take the Σ11{332}{332} as an example, the structure units of the GB in W at 0 and 2400 K are different that part of the atoms near the GB move to the center of the structure units as the temperature increases from 0 to 2400 K, as shown in Figure 5. Meanwhile, the GBEs of the GB in W at 0 and 2400 K are also different, which are 2018 and 2558 mJ·m^−2^, respectively.

### 3.3. The GBs with Lower GBE

Many previous works have reported that the GBs show a strong preference for some special GB planes when the lattice misorientation of the two adjacent grains is specified [7,8,9,54,61,62,63]. It was speculated that these preferred GBs generally have lower GBE, and every preferred GB always has almost the lowest GBE of the GBs with the same lattice misorientation. Our calculations show that for GBs in Mo at 0 K, W at 0 K, W at 2400 K and *β*-Ti at 1300 K, the ∑9{110}{110}, ∑5{310}{310}, and ∑3{211}{211} have almost the lowest GBE of the ∑9 GBs, ∑5 GBs, and ∑3 GBs, respectively. In experiments, the ∑9 GBs of nanocrystalline W [9], the ∑5 GBs of high purity iron [64] and the ∑3 GBs of ferrite [7] showed a strong preference for the {110}{110}, {310}{310}, and {211}{211} planes, respectively. This is in accordance with our calculations. Therefore, it was concluded that the preferred GB are always the one with almost the lowest GBE when the lattice misorientation is specified.

**Figure 6:**
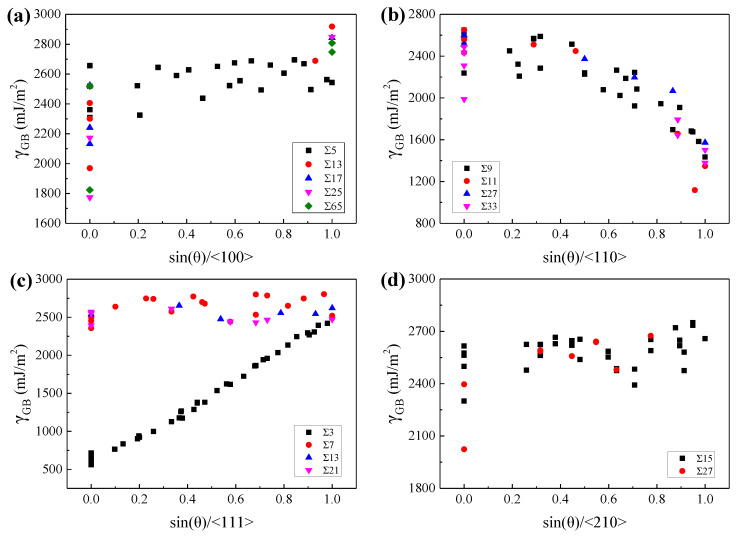
The GBEs of W at 2400 K as a function of sin(*θ*) for GBs with the misorientation axis of (**a**) <100>, (**b**) <110>, (**c**) <111>, and (**d**) <210>, respectively.

**Figure 7:**
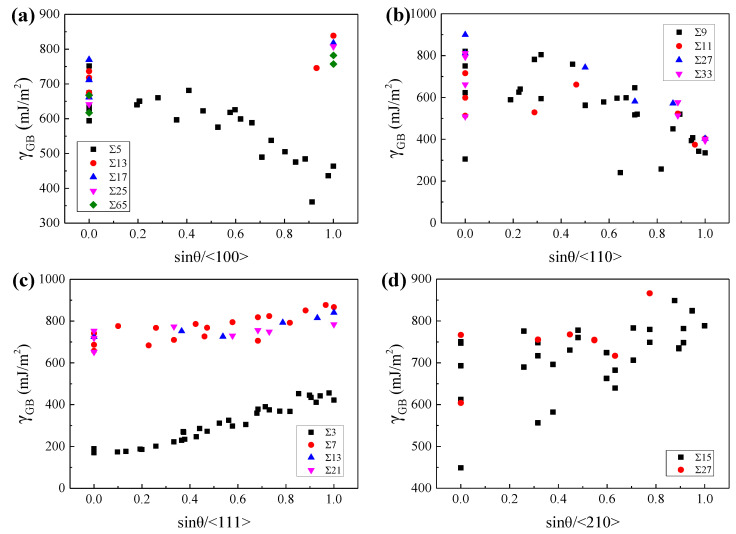
The GBEs of *β*-Ti at 1300 K as a function of sin(*θ*) for GBs with the misorientation axis of (**a**) <100>, (**b**) <110>, (**c**) <111>, and (**d**) <210>, respectively.

To explore the geometric characteristics of the GBs with relatively low GBE, the variations of GBE with sin(*θ*) were drawn for GBs with the same lattice misorientation, as W at 2400 K and *β*-Ti at 1300 K shown in Figure 6 and Figure 7, respectively. It is interesting to note that the variations of GBE with sin(*θ*) are similar for different GB sets which have the same misorientation axis but different misorientation angles. As shown in Figure 6c or Figure 7c, although the ∑3, ∑7, ∑13, and ∑21 GBs have different misorientation angles, the variations of GBE with sin(*θ*) are similar for the four sets of GBs with the same misorientation axis of <111>. Besides, the GB of the lowest GBE is generally one of the GBs with sin(*θ*) = 0 when the lattice misorientation is specified. The GBs with sin(*θ*) = 0 are composed of ATGBs and STGBs, in which the coherent STGBs generally have lower GBE than the ATGBs. According to this finding, it can be predicted that the ∑5, ∑13, ∑17, ∑25, and ∑65 GBs with the misorientation axis of <100> and the ∑3, ∑7, ∑13, and ∑21 GBs with the misorientation axis of <111> will have the lowest GBE at sin(*θ*) = 0 when the GBs are STGBs. The preferred GB planes of these GBs are generally the GB planes of the above-mentioned GBs with the lowest GBE. Thus, the ∑5 and ∑3 GBs will show a strong preference for the {310}{310} and {211}{211} GB planes, respectively. This has been confirmed experimentally in purity iron [64] and ferrite [7].

However, there are two exceptions that the GBs have the lowest GBE when sin(*θ*) is close to 1. The one is the GB set with the misorientation axis of <110> for both W at 2400 K and *β*-Ti at 1300 K, as shown in Figure 6b and Figure 7b. These special GBs have almost the lowest GBE when sin(*θ*) is equal to 1, which are actually the TWGBs with the GB planes of {110}{110}. The low GBE of these special TWGBs is related to the {110}{110} GB planes, which has been discussed in Section 3.1. According to this discovery, it can be predicted that the ∑9, ∑11, ∑27, and ∑33 GBs with the misorientation axis of <110> will have almost the lowest GBE when sin(*θ*) is equal to 1 and the {110}{110} planes will be the preferred GB planes of these GBs. The prediction was experimentally validated that the ∑9 GBs showed a preference for {110}{110} GB plane in nanocrystalline W [9]. The other is the ∑5 (36.9°/<100>) GBs of *β*-Ti at 1300 K. For the ∑5 (36.9°/<100>) GBs, as sin(*θ*) increases from 0 to 1, the GBE of *β*-Ti at 1300 K shows a decreasing trend, which is opposite to the variation trends of GBE for W at 2400 K, as shown in Figure 7a,b. Our calculations show that the ∑5{100}{100} with sin(*θ*) of 1 has almost the lowest GBE of the ∑5 GBs in *β*-Ti at 1300 K. In experiments, the frequently observed textures in near *β*-Ti alloys are generally {100}<100>, {100}<210>, {100}<310>, {111}<112>, {111}<110>, and so on [65,66,67]. The preferred orientation of grains in near *β*-Ti for {100} planes is in accordance with our calculations that the ∑5{100}{100} has the lowest GBE of the GBs with {100}{100} GB planes in *β*-Ti at 1300 K. 

**Figure 8:**
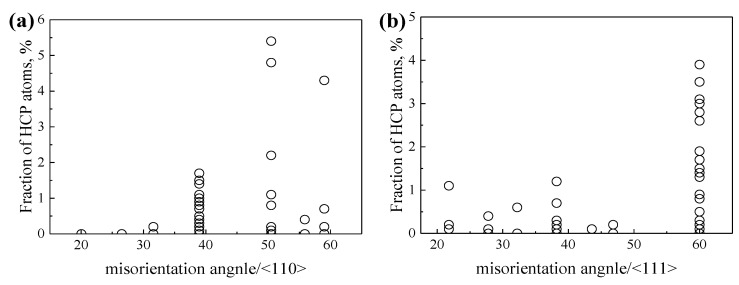
The fraction of HCP atoms as a function of the misorientation angle for GBs with the misorientation axes of (**a**) <110> and (**b**) <111>, respectively.

Compared with the points in Figure 6, the points in Figure 7 are more scattered, which is related to the allotropism of Ti, as discussed in our previous work [55]. Our work early showed that the atomic rearrangements to local hexagonal close-packed (HCP) *α* or hexagonal *ω* phase at GBs may lead to lower GBE of *β*-Ti at 1300 K. The special atomic rearrangement to local HCP *α* phase at GBs of *β*-Ti at 1300 K is related to the lattice misorientation between the two adjacent *β* grains. Figure 8 shows the relationships between the misorientation angles and the formation of HCP *α* phase at GB for GBs with the misorientation axes of <110> and <111>, based on the common neighbor analysis implemented in the software OVITO [68]. It is apparent that the local HCP *α* phase is more likely to form at GBs with the lattice misorientations of 38.9°/<110>, 50.5°/<110>, 59.0°/<110>, and 60.0°/<111>. The later three are in accordance with the experimentally observed lattice misorientations which are 49.5°/<110>, 60.0°/<110>, and 60.0°/<111> [69,70]. Under these special lattice misorientations, it is able to maintain the Burgers orientation relationship (BOR) between the formed GB *α* and the two adjacent *β* grains [70]. Therefore, the special lattice misorientations between the two adjacent *β* grains contribute to the formation of GB *α* phase at the GB and the GB *α* generally has the BOR with both *β* grains.

### 3.4. The Relationships of GBEs between BCC Metals

**Figure 9:**
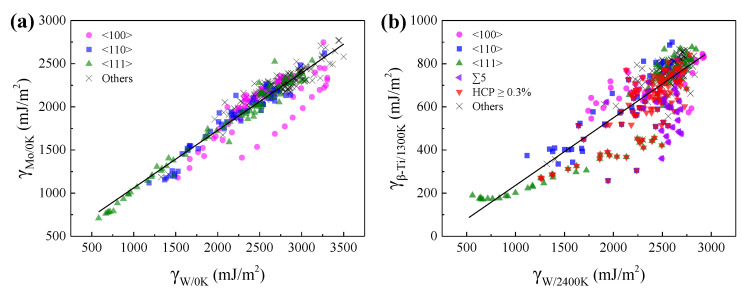
The relationships of GBEs (**a**) between Mo and W at 0 K, and (**b**) between *β*-Ti at 1300 K and W at 2400 K. Every point in Figure 9 corresponds to a GB. The magenta circles, blue squares, olive up triangles, and black crosses in (**a**,**b**) correspond to the GBs with the misorientation axes of <100>, <110>, <111> and others. The violet left triangles and red down triangles in (**b**) correspond to the ∑5 GBs and the GBs with the percentage of HCP atoms no less than 0.3% in *β*-Ti at 1300 K, respectively.

Figure 9 shows the relationships of GBEs between Mo and W at 0 K, and between *β*-Ti at 1300 K and W at 2400 K. As can be seen, there are positive correlations of GBEs between Mo and W at 0 K, and between *β*-Ti at 1300 K and W at 2400 K. The relationship of GBEs between Mo and W at 0 K can be fitted by a linear function and the corresponding slope is 0.67, as shown in Figure 9a. Compared with Figure 9a, although the points in Figure 9b are more scattered, the points can also be roughly fitted by a linear function, and the slope of the line is 0.31. The slopes of the two lines are close to the ratio (0.60) between the *E*_bulk_^2^/*a*_0_^4^ of Mo and W at 0 K, and the ratio (0.35) between the *E*_bulk_^2^/*a*_0_^4^ of *β*-Ti at 1300 K and W at 2400 K, respectively.

The dispersing of the points in Figure 9b is correlated with the allotropism of Ti that the GBEs of *β*-Ti at 1300 K will be further lowered when the atoms at the GB are rearranged to local HCP *α* or hexagonal *ω* phase. As shown in Figure 9b, most of the points are below the fitted line when the proportion of HCP atoms in the GB systems of *β*-Ti at 1300 K is more than 0.3%. In particular, there are several points far below the fitted line, which is related to the special lattice misorientations of 38.9°/<110> and 60.0°/<111>. These special lattice misorientations facilitate the formation of local HCP *α* phase at the GBs of *β*-Ti at 1300 K, which has been discussed in Section 3.3. Besides, most of the points regarding ∑5 GBs are far below the fitted line, which also increase the dispersing of the points in Figure 9b. Although the local HCP *α* and hexagonal *ω* phases were not found in the ∑5 GBs, the GB complexions of these ∑5 GBs are different between *β*-Ti at 1300 K and W at 2400 K. 

## 4. Conclusions

In this study, 405 GBs were constructed for BCC metals and the GBEs were calculated for W at 2400 K and *β*-Ti at 1300 K by MD simulations. Besides, the GBEs of these GBs were also calculated for Mo and W at 0 K by MS simulations for comparison. On the basis of the calculations, the temperature effects on GBEs of W and *β*-Ti, the relationships between GBEs and geometric factors, and the relationships of GBEs between different BCC metals were studied. The major findings are as follows:

1. There is no direct relationship between the GBEs and the ∑ value as a result of the uncertainty of the GB plane, but the relationships between the GBEs and sin(*θ*), where *θ* is the angle between the misorientation axis and the GB plane, can be described by some simple functions for the GBs with the same lattice misorientation. For example, there is a linear relationship between the GBEs and sin(*θ*) for ∑3 GBs. Besides, for the GBs with the {110}{110} GB planes, the GBEs are low and are irrespective of lattice misorientation;
2. The formation of local HCP *α* or hexagonal *ω* phase at GB will lead to lower GBE of *β*-Ti at 1300 K. The local HCP *α* phase is more likely to form at the GBs with special lattice misorientations that the BOR between GB *α* and the two adjacent *β* grains is able to maintain, such as the GBs with the lattice misorientations of 38.9°/<110>, 50.5°/<110>, 59°/<110>, and 60°/<111>;
3. The temperature effects on GBEs of W are limited for most of the GBs. However, for some special GBs, high temperature may result in the GB complexion transitions, accompanied with the change of GBE, such as the Σ11{332}{332};
4. The *γ*-surface method adopted to predict the GB microstructures in ground state shows its drawback in predicting the GB microstructures of TWGBs with relatively high GBE and the GBs with high crystal orientation index of misorientation axis. These special GBs are mostly complexed in GB structures;
5. Generally, the STGB with sin(*θ*) = 0 has the lowest GBE of the GBs with the same lattice misorientation, except for two sets of GBs. The one exception is the GB set with the misorientation axis of <110> that for both W at 2400 K and *β*-Ti at 1300 K, the GB always has the lowest GBE when sin(*θ*) is close to 1. The other exception is the ∑5 GBs that the variation trends of GBE are almost opposite between W at 2400 K and *β*-Ti at 1300 K as sin(*θ*) increases from 0 to 1;
6. The relationships of GBEs between Mo and W at 0 K and between *β*-Ti at 1300 K and W at 2400 K can be roughly described by linear functions and the slopes of the two lines are close to the ratios between the *E*_bulk_^2^/*a*_0_^4^ of Mo and W at 0 K and between the *E*_bulk_^2^/*a*_0_^4^ of *β*-Ti at 1300 K and W at 2400 K, respectively.

The calculated GBEs of W and *β*-Ti at high temperature are an expansion of the GBE database, and the related findings based on these calculations can help to explain and predict the preferred GB planes in BCC metals. Besides, owing to that the GBs show a preference for the GB planes related to lower GBE when the lattice misorientation is specified, the findings of the relationships between GBEs and sin(*θ*) can help to narrow the study scope of GBs with the specific lattice misorientation, which helps to reduce the cost of the systematic studies of GBs.

## Data Availability

All relevant data are available upon request from the authors.
